# Can the different versions of the Shirom-Melamed Burnout Measure be used to measure burnout among healthcare professionals? A systematic review of psychometric properties

**DOI:** 10.1186/s41687-024-00788-8

**Published:** 2024-09-26

**Authors:** Sabrina Figueiredo, Jacques Arrieux, Samia Abdallah, Timothy C. McCall, Ulrich Koch, Eliezer Oliveira

**Affiliations:** 1 Department of Clinical Research and Leadership, School of Medicine and Health Sciences, The George Washington University, 2600 Virginia Ave NW, Suite 300, Office 350, Washington, DC 20037 USA; 2 National Association of County and City Health Officials, Washington, DC USA; 3 Children’s National Hospital, Family Services, Washington, DC USA

**Keywords:** Burnout, Measurement properties, Outcome measures

## Abstract

**Background:**

The prevalence of Occupational Burnout is high among healthcare professionals (HCP). Hence, it is crucial to have robust measures for ascertaining burnout in this population. The Shirom-Melamed Burnout Measure is a prevalent tool used in the diagnosis of burnout, and in the delivery planning of mental health services. The 14-item Shirom-Melamed Burnout *Measure* (SMBM) was developed after a methodological revision of the 22-item Shirom-Melamed Burnout *Questionnaire* (SMBQ). Studies on the psychometric properties of the SMBM and SMBQ exist, but there remains a need for thorough evaluation to assess the methodological quality of individual studies. To address this gap, this systematic review aimed to critically appraise the measurement properties of the different versions of the Shirom-Melamed Burnout Measure/Questionnaire (SMBM/Q) used among healthcare professionals.

**Methodology:**

Four databases (PubMed, CINAHL, PsychINFO, and Scopus) were searched for studies on the psychometric properties of all versions of the SMBM/Q among HCP. The methodological quality of the studies was evaluated using the COSMIN Risk of Bias checklist. Evidence supporting the measurement properties (EMP) of the SMBM was synthesized using the Grading of Recommendations Assessment, Development, and Evaluation (GRADE) approach.

**Results:**

Our final pool consisted of five research articles. One study on the 12-item SMBM was included to estimate content validity, two studies reported on the 14-item SMBM, while the other two employed the 22-item version. Interestingly, each study used the SMBM in a different language, namely English/Hebrew, Chinese, French, Serbian, and Swedish. Structural validity, internal consistency, and construct validity are the SMBM’s most assessed measurement properties. The Hebrew and French versions demonstrated high levels of structural validity and internal consistency, and the remaining reports on validity demonstrated low levels due to methodological flaws.

**Conclusions:**

Per COSMIN guidelines, the SMBM should not be utilized for clinical purposes due to insufficient content validity, but has promising potential with ongoing research. Engaging critical stakeholders for concept elicitation will ensure the relevance, comprehensiveness, and comprehensibility of the PRO items. Likewise, establishing an MIC will allow capturing change over time, which will benefit longitudinal experimental studies.

**Plain English summary:**

Occupational burnout is a significant problem among healthcare professionals, and it is crucial to have a reliable tool to measure it. The Shirom-Melamed Burnout Measure (SMBM) is commonly used to diagnose burnout and plan mental health services. Studies on the psychometric properties of the SMBM exist, but there remains a need for thorough evaluation to assess the methodological quality of individual studies. To address this gap, this systematic review critically appraised the measurement properties of the different versions of the Shirom-Melamed Burnout Measure (SMBM) used among healthcare professionals. Our findings indicate that only a few studies have examined the SMBM, and they have used the tool in different languages. Structural validity, internal consistency, and construct validity are the SMBM’s most assessed measurement properties. We recommend that more research is needed to assess the content validity of the SMBM. We also suggest that critical stakeholders should be involved in the development of the SMBM to ensure that it is relevant, comprehensive, and understandable.

## Introduction

The World Health Organization (WHO) [1] defines burnout as an occupational phenomenon resulting from chronic workplace stress that has not been successfully managed. Among healthcare professionals (HCP), the prevalence of burnout varies significantly. This variation can largely be attributed to an ambiguous definition of burnout, the diversity of healthcare roles often excluded in burnout studies (such as nursing assistants, pharmacists, technicians, etc.), and structural differences in healthcare systems worldwide. Among 248 studies, Canu et al. [2] identified 88 unique burnout definitions. Likewise, Rotenstein et al. [3] identified at least 142 burnout definitions after reviewing 182 longitudinal and cross-sectional studies. Although not classified as an illness or health condition by the WHO, burnout may still necessitate seeking care and contacting health services. Notably, nine countries (Denmark, Estonia, France, Hungary, Latvia, Netherlands, Portugal, Slovakia, and Sweden), have recognized burnout as an occupational disease in contrast with the WHO’s designation as merely an occupational phenomenon.

The burnout prevalence among American physicians ranges from 0 to 80% [3]. Gender differences in burnout prevalence have been reported among HCPs, compared to the general population [4]. Such variations in prevalence may partially stem from the number of diverse outcome measures used to assess burnout [3]. Despite this wide prevalence range, it is agreed upon that the prevalence and awareness of occupational burnout among HCPs reached a peak during the COVID-19 pandemic, which underscores the critical need for robust measures to ascertain burnout in this population [5–9]. In addition, burnout is associated with adverse health effects, decreased quality of care and patient satisfaction, and increased turnover [4, 5].

Several Patient-Reported Outcome Measures (PROMs) are available to capture burnout. The Maslach Burnout Inventory (MBI) [10] is the most widely used measure in burnout research [11] despite fundamental methodological flaws that permeated its development [12]. Other measures include the Burnout Assessment Tool (BAT), Copenhagen Burnout Inventory (CBI), Oldenburg Burnout Inventory (OBI), Pines’ Burnout Measure (BM), Psychologist Burnout Inventory (PBI), Children Services Survey (CSS), Organizational Social Context (OCS), Professional Quality of Life (ProQOL), and the Shirom-Melamed Burnout Measure (SMBM) [13–21]. The latter is a prevalent measure used by the Swedish government in clinical settings and interventional studies [22].

Informed by the Conservation of Resource Theory (COR) [23, 24], Shirom and Melamed conceptualized burnout as a physical, emotional, and cognitive exhaustion [25, 26]. They therefore propose an instrument able to capture this multidimensional construct. Different versions of this instrument exist, implementing varied combinations of subscales and numbers of items [22]. From a total of five existing subscales, four can be used irrespective of specific contexts: (a) emotional exhaustion and physiological fatigue (EPE; sometimes labeled only physiological fatigue), (b) cognitive weariness (CWE), (c) listlessness (LIS), and (d) tension (TES). The fifth subscale—(e) emotional exhaustion in relation to customers—(EEC) applies only to those currently working. All subscales are scored on a 7-point scale ranging from 1 (almost never) to 7 (almost always) with the total score derived by averaging the sum by the number of items [22, 27]. A review of the current literature indicates that this instrument has been used with 12, 14, 18, and 22 items. Toker and colleagues included the 14-item measure in its publication [28]. Without clear specifications of which items are being included, a comparison across studies is not possible.

Studies on the individual psychometric properties of the SMBM report varying results across different populations [21, 22, 29–34]. A recent systematic review of the psychometric properties of the 14-item version of the SMBM exists, but it did not focus on healthcare professionals as their population of interest, and it excluded the other versions of the SMBM currently in use. To address this gap, the present study aims to critically appraise the quality of the measurement properties of all versions of the SMBM used among healthcare professionals by reviewing pertinent findings in the literature.

## Methods

This systematic review followed the Preferred Reporting Items for Systematic Reviews and Meta-Analysis (PRISMA) guidelines. The protocol was registered at the International Prospective Register of Systematic Reviews (registration number CRD42022320711). The methodological quality of individual studies was assessed and evaluated using the COnsensus-based Standards for selecting health Measurement INstruments (COSMIN) checklist, as outlined by Mokkink et al. and updated by Prinsen et al. [35–37].

### Literature search

A comprehensive search strategy for PubMed was developed in collaboration with a University Librarian from the George Washington University using the Terwee Filter. This strategy was adapted to CINAHL, Scopus, WEB of Science, and PsycINFO. No date restriction was applied. Searches were conducted on November 8, 2022, October 30, 2023 and Feb 23, 2024. Search results were exported to Covidence, and duplicates were excluded. The included articles were screened for additional relevant publications.

### Eligibility criteria

#### Inclusion criteria

1. Studies that aimed to evaluate one or more measurement properties and/or the interpretability of any of the versions of the Shirom-Melamed Burnout Measure.
2. Studies where at least 50% of the sample represented healthcare professionals (HCP).

#### Exclusion criteria

1. Studies not written in English, French, or Portuguese.
2. Studies whose full texts were unavailable or unable to be retrieved.
3. Studies correlating SMBM scores with variables other than scores from another outcome measure.

### Selection of studies and data extraction

Two reviewers (SF/JA) independently screened all articles identified by the search. Articles were excluded after a thorough review of titles and abstracts if determined irrelevant. The full texts of the selected articles were retrieved and evaluated against the eligibility criteria. Any uncertainties regarding eligibility were resolved by consensus or by consulting a third investigator (EO). Data was independently extracted by two reviewers (SF/JA) and cross-checked by other investigators (EO). The data extraction form included study identification (i.e., authors, year of publication, journal, and title), burnout PROMs’ characteristics (i.e., name, version, number of items, number of dimensions, dimensions’ names), and statistical methods used for assessing the psychometric properties outcome.

### Methodological quality of individual studies

Two reviewers (SF/JA) independently assessed each study using the COSMIN Risk of Bias checklist [35, 36]. The COSMIN checklist was developed through an international Delphi study and provides a systemic approach for critically appraising studies on PROMs, including design requirements and preferred statistical methods [35, 36].

The COSMIN checklist provides questions for each measurement property covering principles related to study design and statistical tests. Response options range from very good, adequate, doubtful, or inadequate, depending on the quality of the study. The overall quality rating of each single study on a measurement property considers a worst score counts principle. For example, the COSMIN checklist includes five questions to assess the quality of internal consistency. Suppose one study received three ratings as very good, but one as adequate, and one as doubtful. In this case, the overall classification of the internal consistency would be rated as doubtful.

After assessing the methodological quality (risk of bias), we evaluated each study’s results against the updated criteria of good measurement properties. Each result was rated as either sufficient (+), insufficient (–), or indeterminate (?) [37, 38]. The updated criteria for good measurement properties are part of the COSMIN guidelines accessed at https://www.cosmin.nl/.

### Summarizing evidence

The quality of evidence was assessed by two independent reviewers (SF/JA) using the Grading of Recommendations Assessment, Development, and Evaluation (GRADE) approach [39]. This approach evaluates the level of evidence presented based on the risk of bias, consistency, directness, and precision of studies.

The five studies included in this review analyzed the SMBM in a different language, thus are considered a unique version of the measure of interest. Given the linguistic diversity of the SMBM applications in the included studies and the COSMIN guidelines, an overall GRADE rating per measurement property was not provided. Instead, assessments on the quality of evidence for each version of the SMBM were provided. The evidence regarding the measurement properties of each version of the SMBM was compared against the recommendations on PROM selection for use in patient-centered research published by the International Society of Quality of Life (ISOQOL) [40].

## Results

**Fig. 1:**
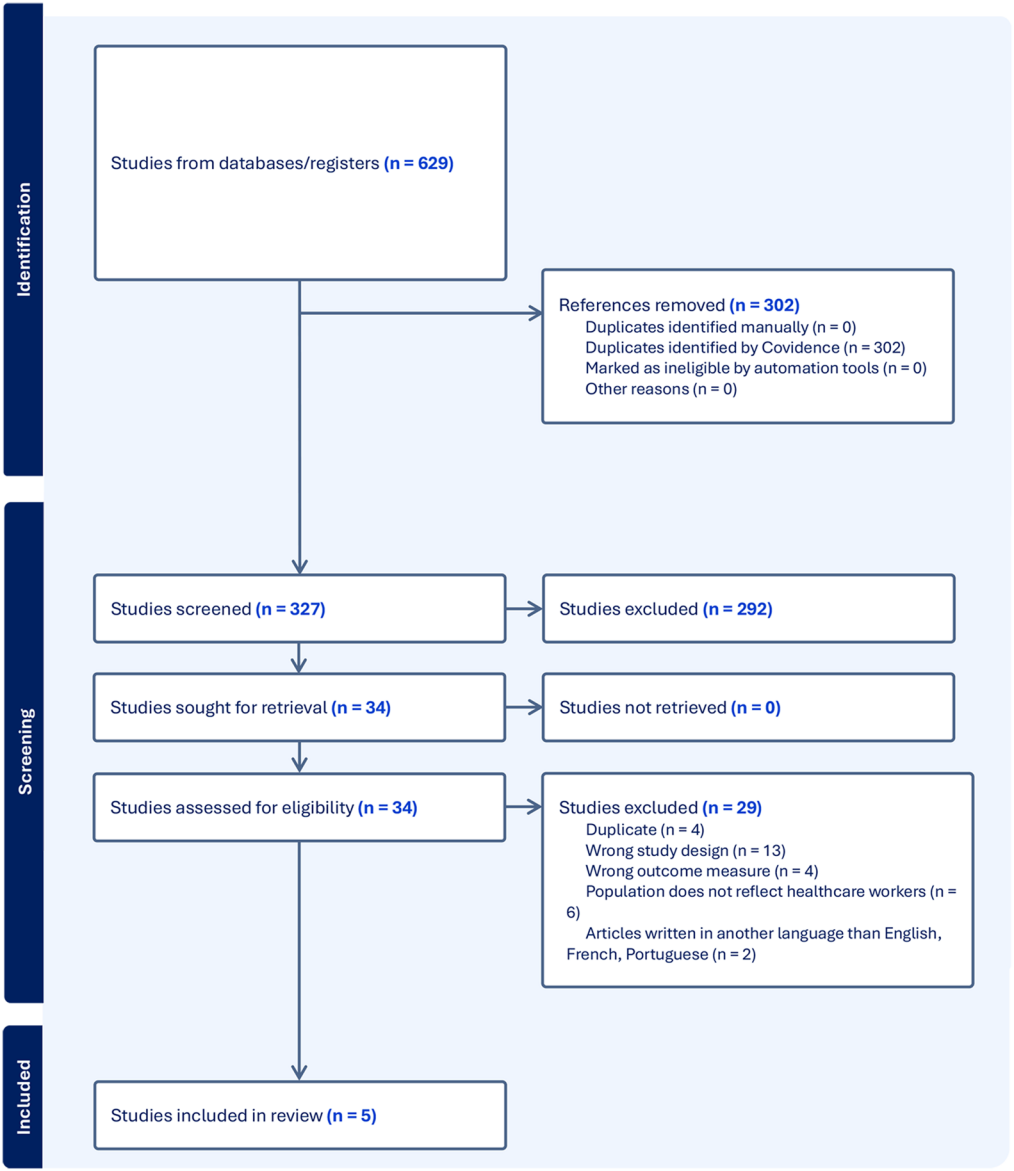
PRISMA diagram of the included studies

The literature search identified a total of 629 articles, and 302 were removed as they were duplicates. Of the remaining 327 studies, 292 were excluded after examination of titles and abstracts for being irrelevant to the topic of interest. The remaining 35 studies were assessed for full-text eligibility, of which four fulfilled our selection criteria. One study was added after revising the included studies’ reference list [31]. Thus, our final pool consisted of five studies [21, 29, 30, 31, 41]. One study from Shirom & Melamed was included, despite the study’s sample being composed of 45% of healthcare professionals [21]. Figure 1 presents the flowchart diagram of the included studies, which evaluated the measurement properties of the SMBM (12 and 14 items) and the SMBQ (22 items) used among healthcare professionals.

Table 1 summarizes the characteristics of the included study populations. One study [30] involved only healthcare workers, whereas the remaining four combined a mix of healthcare and office workers [21, 29, 31, 41]. The samples of the studies were composed predominantly of women, with their average age ranging from 36 to 41. One study used a version with two subscales and 12 items [21]. Two studies (40%) used the 14-item version and three subscales, while the remaining studies (40%) used the 22-item version containing four subscales. Of interest, each study reported on a distinct language in which the SMBM is used, namely English/Hebrew, French, Chinese, Serbian, and Swedish.

**Table 1:** Characteristics of the included study populations

Study	Number of dimensions and number of items	Population			Instrument characteristics		
		*N*	Age mean (SD), yr.	Gender % female	Setting	Country	Language
Shirom and Melamed [21]	2:12	S1: 198	39 (9)	66	Healthcare	Israel	Hebrew
		S2: 236	36 (10)	59	Office		
Sassi and Neveu [29]	3:14	S1: 203	39 (10)	72	Healthcare	France	French
		S2: 214	41 (11)	65	Office		
Qiao and Schaufeli [30]	3:14	717	NR	100	Healthcare	China	Chinese
Jocic et al. [41]	4:22	514	NR	NR	Healthcare workers	Serbia	Serbian
Lundgren-Nilsson et al. [31]	4:22	319	42 (9.5)	69	Healthcare and social workers	Sweden	Swedish

*NR* not reported

Structural validity, outlined in the COSMIN guideline as the degree to which the scores of an HR-PRO instrument are an adequate reflection of the dimensionality of the construct to be measured [36, 37], was assessed in all included studies [21, 29, 30, 31, 41]. Internal consistency, reflected by the degree of interrelatedness among the items [36, 37], was the second most assessed property (*n* = 4) [21, 29, 30, 41]. Construct validity, which is the degree to which the scores of an HR-PRO instrument are consistent with hypothesis based on the assumption that the HR-PRO instrument validly measures the construct to be measured [36, 37] was the third most commonly assessed measurement property, being assessed in three studies [21, 30, 41]. Two studies reported on content validity [21, 41], defined as the degree to which the content of an HR-PRO instrument is an adequate reflection of the construct to be measured [36, 37]. No studies reported on reliability (the proportion of the total variance in the measurements which is due to true difference between patients [36, 37]), measurement error (the systematic and random error of a patient’s score that is not attributed to true changes in the construct to be measured [36, 37]), and responsiveness (the ability of an HR-PRO to detect change over time in the construct to be measured [36, 37]) in our population of interest. In addition, we did not report on criterion validity, defined as the degree to which the scores of an HR-PRO instrument are an adequate reflection of the gold standard [36, 37], due to the lack of a gold standard for measuring burnout.

Table 2 summarizes the methodological quality and the results of each study per measurement property. Content validity of the SMBM-12 items published by Shirom and Melamed [21], was rated as inadequate due to the lack of a qualitative method to identify relevant PROM items. Likewise, the content validity of the Serbian version [41] was rated as doubtful because the patient interview methodology is not clearly described. As for structural validity, two studies (40%) received very good quality [21, 29] ratings due to statistical methods and study design. Of the three remaining studies (60%), one [31] received an adequate quality rating due to the use of exploratory factor analyses and the uncertainty around the chosen Rasch Model. The other two studies [30, 41] were rated doubtful for not describing rotation methods.

**Table 2:** Methodological quality and results of studies on measurement properties

PROM characteristics		Content validity		Internal structure						Remaining measurement properties				
PROM (ref)	Language	PROM develop	Content validity	Structural validity			Internal consistency			Reliability	Measure error	Construct validity		Responsiveness
				*n*	Meth Qual	Result (rating)	*n*	Meth Qual	Result (rating)			*n*	Meth Qual	Result (rating)
12 items														
Shirom and Melamed [21]	Hebrew	I	I	434	VG	Original model 2 factors, 12 items CFI = 0.94 & RMSEA = 0.08 (–)	434	VG	PF: 0.92 CW: 0.92 Cronbach alpha > 0.7; but the criteria for structural validity was not met as sufficient (?)			434	VG	Results not in accordance with the hypothesis (–)
						Revised model								
						2 factors, 11 items CFI = 0.97 RMSEA = 0.05 (+)			Cronbach alpha > 0.70 (+)					
14 items														
Sassi and Neveu [29]	French			417	VG	3 factors CFI = 0.957 (+)	417	VG	PF = 0.87 CW = 0.93 EE = 0.84					
									Cronbach alpha > 0.70 (+)					
Qiao and Schaufeli [30]	Chinese			717	D	1 factor, 14 items CFI = 0.81 & RMSEA = 0.15 (–)	717	D	PF = 0.9 CW = 0.85 EE = 0.75			717	D	No hypothesis defined (?)
									Cronbach alpha > 0.7; but the criteria for structural validity was not met as sufficient (?)					
						3 factors, 14 items CFI = 0.90 & RMSEA = 0.10 (–)								
						3 factors revised CFI = 0.93 & RMSEA = 0.09 (–)								
22 items														
Jocic et al. [41]	Serbian		D	514	D	CFI < 0.95 (–)	514	D	PF: 0.89 CW: 0.95 EE: 94 TE: 0.91 LIS: 0.84 Cronbach alpha > 0.7; but the criteria for structural validity was not met as sufficient (?)			514	D	No hypothesis defined (?)
Lundgren-Nilsson et al. [31]	Swedish			319	A	1 factor CFI = 0.94 RMSEA = 0.17 (–)								
						4 factors, 22 items CFI = 0.97 RMSEA 0.133 (?)								
						Revised 18 items Rasch Infit and outfit mean square = NR Z-standardized values = NR (?)								

Methodological Quality = Very Good (VG), Adequate (A), doubtful (D), inadequate (I); Result Rating = sufficient (+), insufficient (–), inconsistent (±), or indeterminate (?). *PF* physical fatigue, *CW* cognitive weariness, *EE* emotional exhaustion, *TE* tension, *LIS* listlessness, *NR* not reported

When the structural validity results of these five studies were compared against the criteria for good measurement properties, most of the studies (*n* = 3) were rated as insufficient [30, 31, 41]. Among studies assessing internal consistency (*n* = 4) [21, 29, 30, 41], the highest quality rating (very good) was given to two studies (50%) [21, 29]. The other two studies received quality ratings of doubtful [30, 41] due to methodological flaws. When compared against the criteria for good measurement properties—the internal consistency results of these four studies were either deemed sufficient [21, 29] or indeterminate [30, 41]. Regarding construct validity, the methodological quality of studies (*n* = 3) ranged from very good [21] to doubtful [30, 41]. When comparing the results of these studies against the criteria for good measurement properties they were either rated as insufficient [21] or indeterminate [30, 41].

Table 3 outlines the evidence level for each SMBM version. The five studies included in this review analyzed the SMBM in various languages, featuring different numbers of subscales and items. This heterogeneity prevented the provision of a pooled GRADE rating, thus ratings for each property for each version of the SMBM were provided. According to the GRADE guidelines [39], there are four levels for the quality of evidence: very low, low, moderate, and high. Among the evaluated versions of the SMBM, none reported on all measurement properties, and 66% (10/15) of the measurement properties were supported by low levels of evidence. The Serbian version of SMBM-22 items [41] had low levels of evidence supporting internal consistency, structural, and construct validity, whereas the Swedish version [31] had moderate levels of evidence. The Chinese version of SMBM-14 items [30] demonstrated low evidence supporting structural validity, internal consistency, and construct validity. There was high evidence supporting the structural validity and internal consistency of the Hebrew version of SMBM-12 items [21] and the French version of SMBM-14 items [21, 29].

**Table 3:** Level of evidence per patient-reported outcome measure and measurement property

PROM characteristics		Content validity		Internal structure			Remaining measurement properties			
PROM measure/study	Language	PROM develop	Content validity	Structural validity	Internal consistency	Cross cultural validity	Reliability	Measure error	Construct validity	Responsivenes
12 items										
Shirom and Melamed [21]	Hebrew	Low	Low	High	High				Low	
14 items										
Sassi and Neveu [29]	French			High	High					
Qiao and Schaufeli [30]	Chinese			Low	Low				Low	
22 items										
Jocic et al. [41]	Serbian		Low	Low	Low				Low	
Lundgren-Nilsson et al. [31]	Swedish			Moderate						

## Discussion

Burnout among healthcare professionals remains a pressing concern for healthcare delivery systems. Burnout is largely characterized by subjective experiences that vary among individuals. Due to the nature of this construct, it is best captured using PROMs to capture the nuances and degrees of burnout that other measures cannot. Several such measures exist, yet it is uncertain whether they meet the ISOQOL’s minimum standards [40] for selecting PROMs for use in patient-centered outcomes research, as well as the COSMIN recommendation guidelines [35, 36, 37]. The purpose of this systematic review was to critically appraise the measurement properties of the different versions of the Shirom-Melamed Burnout Measure/Questionnaire used among healthcare professionals. Five studies were included: one used the 12-item, two used the 14-item SMBM, and the remaining two employed the 22-item version. Of interest, each study used the SMBM in a different language, namely Hebrew, Chinese, French, Serbian, and Swedish, and therefore were treated as independent versions of the SMBM.

The SMBM is a prevalent tool used by the Swedish Health System as a diagnostic tool for burnout and planning the delivery of mental health services. In Sweden, the SMBM is regarded as the state-of-the-art measure and is the most frequently used in intervention studies. Consequently, it is unsurprising that Swedish researchers have contributed a significant number of measurement studies concerning the SMBM. That said, two of these papers [22, 34] were not included in this review due to our eligibility criteria and focus on healthcare professionals. Consistent with COSMIN guidelines, observational studies estimating the association between burnout and physiological variables such as diabetes, cardiovascular function, and salivary cortisol were excluded as they do not constitute measurement studies [28, 42–47]. In addition, these initial papers published by Shirom and Melamed failed to adequately describe the measure development and characteristics, including the original language and the population.

The most important measurement property in an instrument is content validity, followed by structural validity and internal consistency, for the latter two properties assess the internal structure of an instrument. The remaining measurement properties, such as reliability, measurement error, hypothesis testing for construct validity, and responsiveness, rank third in importance and their purpose is to evaluate the quality of an instrument as a whole [35–37]. Our findings suggest that the content validity of the SMBM is inadequate, since it was not developed by engaging a diverse group of healthcare professionals (i.e., the population of interest) during its development phase. Therefore, it needs to be clarified whether the SMBM items are relevant, comprehensive, and comprehensible. The SMBM’s internal structure was assessed in five studies included in this review [21, 29, 30, 31, 41]. The Hebrew and French versions demonstrated high evidence [21, 29], and the Swedish version showed moderate evidence levels [31]. However, the remaining reports on the internal structure of the SMBM [30, 41], as well as the three studies reporting on hypothesis testing for construct validity, demonstrated low levels of evidence due to methodological flaws. As a result, we cannot determine how the SMBM scores can be compared against other instruments. While the Hebrew, French, and Swedish versions of the SMBM include items adequately combined in subscales to measure the different domains of burnout, there is uncertainty about how the items of the Chinese and Serbian versions relate among themselves.

Furthermore, there is no report on the reliability, measurement error, and responsiveness of the SMBM in our population of interest. Therefore, there is uncertainty about whether similar scores would be obtained if the respondent’s condition is stable. On the other hand, the lack of information on responsiveness implies uncertainty on whether the instrument can detect change over time. Likewise, the absence of an established minimal important change (MIC) impedes adopting the SMBM in longitudinal experimental studies. Another significant concern pertains to using a 7-point Likert scale to calculate a total score for the SMBM by averaging the responses by the number of items [21]. All reviewed studies that employed confirmatory factor analysis (CFA)indicate that burnout measured by the SMBM is not unidimensional. Therefore, using a total score, as proposed by Shirom & Melamed [27], is not recommended. Instead, it is recommended that scores for individual subscales should be calculated unless the measure was developed using Item Response Theory, such as Rasch Analysis.

After a systematic review of measurement properties is completed, the COSMIN checklist suggests a definitive recommendation be made. According to our findings, the SMBM should not be recommended because PRO items might need to be more relevant, comprehensive, and comprehensible. It is important to acknowledge and consider that the SMBM was developed prior to the establishment of the COSMIN checklist. This may account for its focus on other psychometric properties related to the internal structure of the tool. Additionally, the SMBM also does not meet the International Society of Quality of Life (ISOQOL) minimum standards for selecting PROM for use in patient-centered outcomes research.

Likewise, a systematic review of the psychometric properties of the 14-item version of the SMBM in a general population reported low levels of evidence. Despite similar results, our eligibility criteria differed greatly:

1. Studies using all versions of the SMBM and published in English, French, or Portuguese were included. Shoman et al. [48], included only the 14-item version published in English.
2. Our population of interest was limited to healthcare professionals, whereas Shoman et al. [48] reported on the general population.
3. Adhering to the COSMIN guidelines, our analysis excluded studies that did not aim to evaluate measurement properties of the SMBM such as observational studies investigating or estimating the association between burnout and physiological variables. Conversely, such reports were included in the work conducted by Shoman et al. [48].

As a result, the final pool of included articles in our review differs from what was reported by Shoman and colleagues. We included five studies, while they included seven. Only one reference [21] overlaps in these two reviews.

Additional systematic reviews of psychometric properties reporting on seven other burnout measures also found to have a very low quality of evidence on content validity of the Maslach Burnout Inventory, Burnout Measure, and the Psychologists Burnout Inventory [10, 16, 17]. The Copenhagen Burnout Inventory (CBI) was developed in 2005 [14] to address cultural gaps of the Maslach Burnout Inventory (MBI), and the Burnout Assessment Tool (BAT) were the only PROMs demonstrating a moderate quality of evidence on content validity [48, 49].

The CBI encompasses three sub-dimensions: personal burnout, work-related burnout, and client-related burnout [14]. The three subscales of the questionnaire were designed to be applied in different domains. The questions on personal burnout were formulated as a generic scale. The work-related burnout questions relate to paid work. The measure has been translated into eight languages and is publicly available. Another promising measure in the field is the Burnout Assessment Tool (BAT) [13]. Published by Schaufeli in 2020, the BAT was developed to address methodological shortcomings of the Maslach Burnout Inventory [10] with a 23-item and a 12-item version measuring four burnout dimensions. In 2023, Redelinghuys and Morgan investigated score interpretation, convergent validity, and measurement invariance of the BAT in Australia, Netherlands, South Africa, and the United States using the Rasch measurement model [50]. Despite their promise, the CBI and BAT have psychometric properties that require further evaluation. Of interest, Lundgren-Nilsson et al. analyzed the SMBM 22-item using Rasch Analysis after CFA failed to confirm adequate ratings [31]. Following the removal of four items from the Tension subscale, the authors reported the revised 18-item version satisfies modern measurement standards. However, this version also necessitates further testing.

Burnout among healthcare workers has escalated to epidemic levels. There remains a pressing need for effective and efficient interventions. However, the lack of a sound and reliable measurement tool impedes the design of robust randomized controlled trials. The SMBM version presented in 2006 [21] is promising given its domains and number of items [22]. Revision of the SMBM is warranted to address the measurement issues related to inadequate content validity. In a similar approach, Carriere et al. [51] conducted a qualitative study with six rounds of focus groups to improve the Patient and Observer Scar Assessment Scale (POSAS) [52] through feedback from individuals living with scars.

Our review underscores the necessity for further research aimed at improving the measurement of burnout from the perspective of HCP.

## Conclusion

Informed by the Conservation of Resources Theory, the SMBM possesses a strong theoretical basis. However, according to the COSMIN guidelines, this instrument cannot be recommended for use due to inadequate content validity. Given its promising potential, the SMBM warrants revision to improve its content validity by engaging critical stakeholders for concept elicitation and cognitive interviews. Both processes will ensure the relevance, comprehensiveness, and comprehensibility of the PRO items. The revision of the SMBM will also benefit from estimating the remaining measurement properties for which no reports were retrieved.

## Strength and limitations

Informed by COSMIN guidelines [35–37], this study employed a systematic approach to review and evaluate the measurement properties of the SMBM among HCP. The comprehensive search strategy used a validated filter for studies on measurement properties and included articles in English and French from four different databases over 15 years. In addition, the review was conducted and reported according to the PRISMA guidelines and was registered a priori to mitigate publication and reporting bias. The findings of this study, however, should be interpreted with caution as our target population was limited to healthcare professionals. Recent studies have assessed the reliability and validity of the SMBM in different populations. For instance, Gerber et al. [33] investigated the SMBM measurement properties in students, Schilling et al. [32] in police officers, and Almen & Jansson [22] and Sundstrom et al. [34] in a general Swedish sample. Another limitation is that we only found one article per measurement version, which yielded heterogeneous findings that precluded a comprehensive overall GRADE assessment [39]. Finally, the low levels of evidence can be a function of poor and inadequate reporting rather than fundamental methodological issues.

## Data Availability

The data that support the findings of this study are available from the corresponding author, SF, upon reasonable request.
